# Protective Effect of Flavonoids from *Ziziphus jujuba cv. Jinsixiaozao* against Acetaminophen-Induced Liver Injury by Inhibiting Oxidative Stress and Inflammation in Mice

**DOI:** 10.3390/molecules22101781

**Published:** 2017-10-20

**Authors:** Weizhen Huang, Yongjie Wang, Xiaoyan Jiang, Yueyue Sun, Zhongxi Zhao, Siying Li

**Affiliations:** 1 School of Pharmaceutical Sciences, Shandong University, 44 West Wenhua Road, Jinan 250012, Shandong, China; huangwzh93@163.com (W.H.); wyj201710@163.com (Y.W.); jiangxiaoyan1121@163.com (X.J.); 13021717075@163.com (Y.S.); 2 Shandong Engineering & Technology Research Center for Jujube Food and Drug, 44 West Wenhua Road, Jinan 250012, Shandong, China; 3 Shandong Provincial Key Laboratory of Mucosal and Transdermal Drug Delivery Technologies, Shandong Academy of Pharmaceutical Sciences, 989 Xinluo Street, Jinan 250101, Shandong, China; 4 Department of Pathology and Pathophysiology, School of Basic Medicine, Shandong University, 44 West Wenhua Road, Jinan 250012, Shandong, China

**Keywords:** *Ziziphus jujuba cv. Jinsixiaozao*, Flavonoids, hepatoprotective, antioxidant activity, Nrf2, anti-inflammation, NF-κB

## Abstract

This study was aimed to investigate the chemical composition, antioxidant activities and hepatoprotective effect of flavonoids from *Ziziphus jujuba cv. Jinsixiaozao* (ZJF). The composition of ZJF was analyzed by high performance liquid chromatography (HPLC) and Liquid chromatography–mass spectrometry (LC–MS), and antioxidant properties were investigated by biological assays in vitro. The hepatoprotective activity of ZJF was evaluated in acetaminophen (APAP)-treated BALB/c mice. Results indicate that ZJF displayed significant antioxidant capacity. Pretreatment with ZJF significantly decreased APAP-elevated serum alanine aminotransferase (ALT), aspartate aminotransferase (AST), alkaline phosphatase (ALP) and total bilirubin (TB). Activities of superoxide dismutase (SOD) and glutathione peroxidase (GSH-Px) were enhanced with ZJF administration, while malondialdehyde (MDA) level and glutathione (GSH) depletion were reduced. Meanwhile, ZJF reversed the suppression of nuclear factor erythroid 2-related factor 2 (Nrf2) nuclear translocation, and up-regulated the protein expression of NAD(P)H: quinone oxidoreductase 1(NQO1) in liver damage mice. Furthermore, ZJF attenuated APAP-induced inflammatory mediator production, such as nitric oxide (NO), tumor necrosis factor-α (TNF-α), interleukin-6 (IL-6) and interleukin-1β (IL-1β). Expression of p65 showed that ZJF dampened nuclear factor-κB (NF-κB) activation. The results strongly indicate that the hepatoprotective role of ZJF in APAP-induced hepatotoxicity might result from its induction of antioxidant defense via activation of Nrf2 and reduction of inflammation via inhibition of NF-κB.

## 1. Introduction

Nowadays, liver diseases remain one of the major threats to public health, as the organ plays a pivotal role in the metabolism of endogenous and exogenous substances. Drug-induced liver injury has become the main reason of acute liver injury, especially acetaminophen (APAP)-induced hepatotoxicity [1,2]. APAP is a widely used drug for its analgesic and antipyretic properties, but overdose causes severe hepatotoxicity and nephrotoxicity [3,4]. It is widely accepted that APAP is metabolized in the liver primarily by glucuronidation and sulfation pathways at therapeutic doses, and only a small fraction is converted by liver cytochrome P450 into a highly reactive metabolite product named N-acetyl-p-benzoquinone imine (NAPQI), which depleted glutathione (GSH) [5,6]. At a toxic dose, GSH is exhausted, and excessive NAPQI will covalently bind to cellular proteins, which leads to oxidative stress, inflammation, lipid peroxidation, mitochondrial dysfunction and centrilobular necrosis [1,7,8,9]. Therefore, there is an urgent need for complementary and alternative medicines for the preventive treatment of APAP toxicity. Studies on herbal medicines indicate that phytochemicals possessing strong antioxidant and free radical scavenging abilities as well as anti-inflammatory actions can be developed for therapeutically effective agents for the treatment of liver diseases [10].

The fruit of *Zizyphus jujuba* Mill, belonging to the Rhamnaceae family, is a historically prescribed herb in folklore medicine for the treatment of various diseases. Extensive phytochemical studies in recent years have revealed that jujube contains many functional constituents such as triterpenic acid [11,12], flavonoids [13], phenolic acids [14,15], polysaccharides [16,17], and amino acids [18], which have been shown to be responsible for various pharmacological effects including antiproliferation of cancer cells [19,20,21], regulation of immune function [16], anti-inflammatory [22], antiobesity [23], antioxidant [24,25], hepatoprotective [26], and gastrointestinal protective activities [27]. As secondary metabolites, flavonoids possess multiple pharmacological actions such as antioxidant [28], anti-inflammatory [29], and antimicrobial activities [30], which makes it a potential agent for hepatic protection. Bifendate is a commonly used drug for the treatment of drug-induced liver injury, and is often selected as a positive control drug for the evaluation of potential hepatoprotective agents [31,32]. Therefore, in this study, we selected bifendate as a control drug for evaluating the hepatoprotective activity of flavonoids from *Ziziphus jujuba cv. Jinsixiaozao* (ZJF).

It has been reported that most genes overexpressed in the inflammatory response are controlled by the nuclear factor kappa B (NF-κB) [33]. The genes encoding antioxidant proteins and phase II detoxifying enzymes are modulated by a transcription factor nuclear erythroid 2-related factor 2 (Nrf2). Thus, to evaluate the signaling pathways involved in the protective role of ZJF against APAP-induced liver injury, we further investigated the roles of Nrf2 and NF-κB which may be the potential molecular mechanism accounting for the antioxidant and anti-inflammatory activities of ZJF.

To the best of our knowledge, there is no systematical report available on the chemical composition, antioxidant, and hepatoprotective effects of ZJF. Hence, the present study focused on assessing the chemical composition of ZJF and its antioxidant activity. Furthermore, the underlying mechanism of its protection against APAP-induced hepatotoxicity was investigated by evaluating its antioxidant and anti-inflammatory effects.

## 2. Results

### 2.1. Content Assay and Chemical Composition Analysis of ZJF

It is necessary to reveal the chemical composition to make a rational assessment on the biological activity of ZJF. Limited research has reported on the identification of flavonoids from *Ziziphus jujuba*. Most of the previous studies focused on the assay of total flavonoid content by different colorimetric assay methods [24,34,35]. In our study, total flavonoid content of ZJF was determined to be 40.85% rutin equivalent via ultra-violet (UV) spectrophotometer.

**Figure 1:**
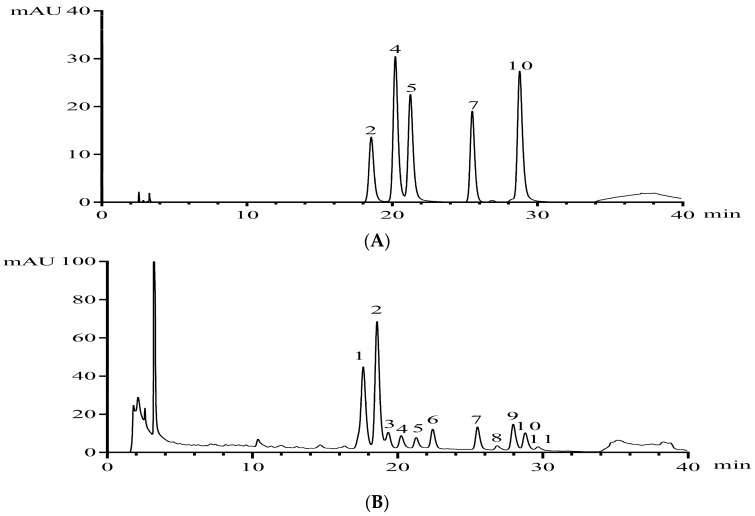
A representative high performance liquid chromatography (HPLC) chromatogram acquired at 350 nm of (**A**) flavonoid standards (**B**) flavonoids in flavonoids from *Ziziphus jujuba cv. Jinsixiaozao* (ZJF). Peaks were tentatively identified as (**1**) quercetin-3-*O*-robinobioside, (**2**) quercetin-3-*O*-rutinoside, (**3**) Unidentified, (**4**) quercetin-3-*O*-galactoside, (**5**) quercetin-3-*O*-glucoside, (**6**) kaempferol-3-*O*-robinobioside, (7) Kaempferol-3-*O*-rutinoside, (**8**) Unidentified, (**9**) Quercetin-3-*O*-arabino-rhamnoside, (**10**) Quercetin-3-*O*-rhamnoside, (**11**) Quercetin-3-*O*-xyloso-rhamnoside.

The chemical compositions in ZJF were analyzed by HPLC-diode array detector (DAD) and liquid chromatography–electron spray ionization-mass spectrometry (LC-ESI-MS) analyses. A representative chromatogram was obtained at 350 nm (Figure 1) and the results of tentative identification are shown in Table 1. Two types of flavonol derivatives with the MS/MS fragments at around *m*/*z* 301 and 285 were characteristic of quercetin and kaempferol derivatives, respectively. Based on the retention time, UV adsorption values and MS spectral data comparing with the authentic commercial standards, peaks 2, 4, 5, 7, 10 were identified as quercetin-3-*O*-rutinoside, quercetin-3-*O*-galactoside, quercetin-3-*O*-glucoside, kaempferol-3-*O*-rutinoside, and quercetin-3-*O*-rhamnoside, respectively (Table 1). Peak 1 at 17.63 min yielded a deprotonated molecular [M−H]^−^ ion at *m*/*z* 608.9. The fragment ion of *m*/*z* 300.2 (quercetin moiety) was generated by loss of two sugar units. UV spectrum showed λ_max_ at 252 and 353 nm, suggesting that it was a quercetin glycoside and tentatively identified as quercetin-3-*O*-robinobioside by examining the two known compounds, quercetin-3-*O*-rutinoside (*t*_R_ = 18.58, identified by comparison with the standard) and quercetin-3-*O*-robinobioside reported previously in the published data [13]. The mass spectrometric characterization of compounds at 27.96 and 29.67 min provided evidences for the presence of two quercetin pentosides, with *m*/*z* 579.5 as the pseudo-molecular ion. By consulting the current information with those in literature [13,25], the analytes were tentatively identified as quercetin-3-*O*-arabino-rhamnoside and quercetin-3-*O*-xyloso-rhamnoside, respectively. A peak at 22.41 min showed a precursor ion at *m*/*z* 593.4. Owing to a combined loss of two sugar units, its mass spectrometry/ mass spectrometry (MS/MS) spectrum gave a fragment ion at *m*/*z* 285 corresponding to kaempferol glycoside derivatives. In addition, UV spectrum of this compound with λ_max_ at 260 and 350 nm was typical of flavonol derivatives. By examining the known kaempferol glycoside in jujube [13,25]. Kaempferol-3-*O*-robinobioside and kaempferol-3-*O*-rutinoside were consistent with the above data. Furthermore, the peak at 25.50 min was straightway confirmed as kaempferol-3-*O*-rutinoside by comparing with its standard. From these results and cited studies, peak 6 was tentatively identified as kaempferol-3-*O*-robinobioside. The UV/vis absorptions of peak 3 and 8 were around 250 and 350 nm, which were characteristic absorption wavelengths of flavonoids, the structures of which could only be needed to be further elucidated.

### 2.2. In Vitro Antioxidant Activity of ZJF

**Figure 2:**
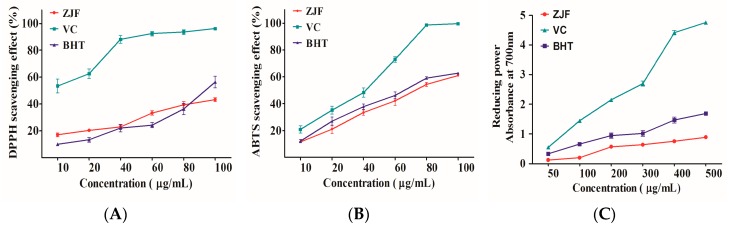
Antioxidant activity of ZJF: (**A**) 1,1-diphenyl-2-picrylhydrazyl (DPPH) radical scavenging assay; (**B**) 2,2′-azino-bis(3-ethylbenzothiazoline-6-sulfonic acid) (ABTS) radical scavenging assay and (**C**) Ferric Reducing Power assay (FRAP). Data are shown as mean ± Standard Error of Mean (SEM) (*n* = 3).

Flavonoids have been postulated to exert a positive effect on the prevention of chronic diseases due to its antioxidant activity [36]. Free radical scavenging capacity is an important feature of antioxidants, since free radicals can initiate lipid peroxidation, cause DNA strand breaks, form protein adducts, and oxidize molecules in biological membranes and tissues. Free radical scavenging capacity in vitro has been evaluated by various methods under different conditions. 1,1-diphenyl-2-picrylhydrazyl (DPPH) and 2,2′-azino-bis(3-ethylbenzothiazoline-6-sulfonic acid) (ABTS) free radical scavenging assays are more convenient and rapid indirect methods. Thus, DPPH and ABTS radical scavenging assays have been widely used to assess the capacity of scavenging free radicals of various complex mixtures of plants [34]. In the present study, butylated hydroxytoluene (BHT) and ascorbic acid (VC) were used as the reference commercial antioxidants. The results are shown in Figure 2. In these three assays, VC showed significantly higher radical scavenging activity and reducing power compared with ZJF and BHT. There was no significant difference between the antioxidant capacities of ZJF and BHT. Furthermore, the results from these three test systems reveal that ZJF had the relatively strong antioxidant activity.

### 2.3. Biochemical Indicators of Liver Function

#### 2.3.1. Levels of Alanine Aminotransferase, Aspartate Aminotransferase, Alkaline Phosphatase and Total Bilirubin in Serum

**Figure 3:**
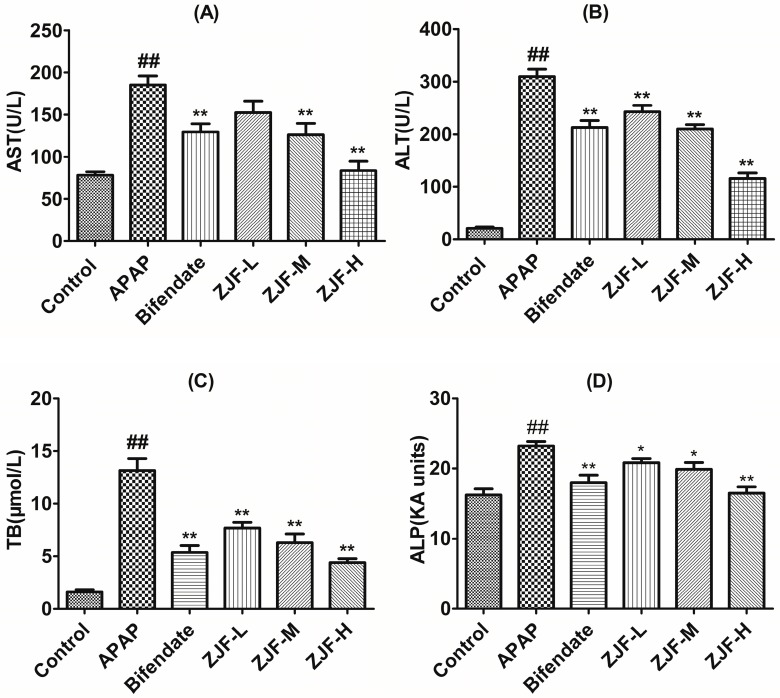
Preventive effects of different doses of ZJF on the activities of serum aspartate aminotransferase (AST), alanine aminotransferase (ALT), total bilirubin (TB), and alkaline phosphatase (ALP) in APAP-induced liver damage mice. (**A**) level of AST; (**B**) level of ALT; (**C**) level of TB; (**D**) level of ALP. Values are presented as Mean ± SEM, *n* = 6. ## *p* < 0.01 compared to control group. * *p* < 0.05 and ** *p* < 0.01 compared to APAP treated group.

It is well known that aspartate aminotransferase (AST), alanine aminotransferase (ALT), total bilirubin (TB), and alkaline phosphatase (ALP) are sensitive serum biochemical markers for early acute hepatic damage. To evaluate the hepatoprotective activity of ZJF against the APAP-induced hepatic injury, the serum levels of AST, ALT, TB and ALP were analyzed by commercial reagent kits. Results of the liver function detection are showed in Figure 3. The APAP-intoxicated group exhibited a notable elevation of AST, ALT, TB, and ALP levels in serum compared to the control group (*p* < 0.01). In contrast, the levels of ALT, TB, and ALP were dose-dependently reduced in low-, medium-, high-dose ZJF pretreatment groups (*p* < 0.05 or *p* < 0.01). AST levels showed a significant decrease at the dose of 400 mg/kg, which was close to the control. It was confirmed that ZJF could prominently ameliorate hepatoxicity induced by APAP.

#### 2.3.2. Measurement of Malondialdehyde, Glutathione and Activity of Antioxidant Enzymes in the Liver

**Figure 4:**
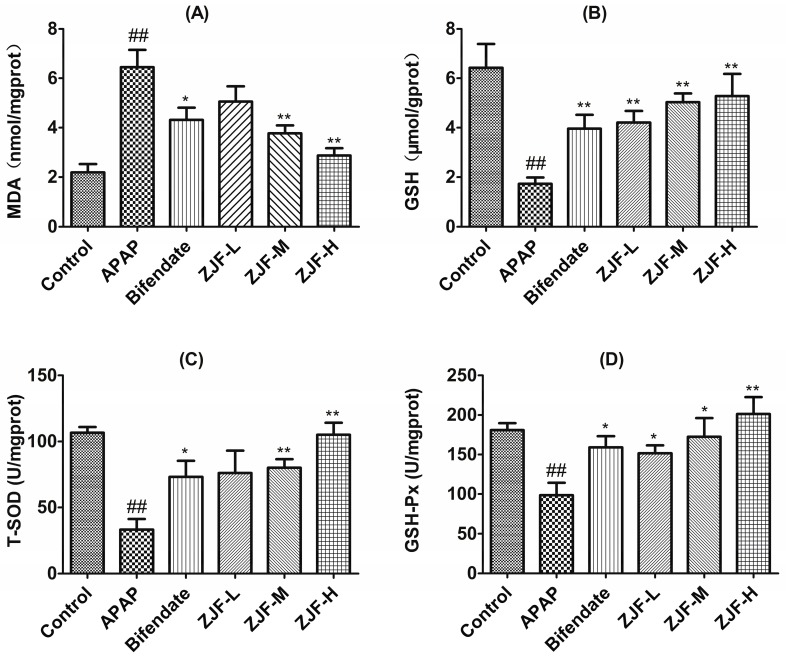
Effects of ZJF on the oxidative damage markers in liver homogenate after APAP overdose. (**A**) Hepatic level of malondialdehyde (MDA); (**B**) Hepatic glutathione (GSH) contents; (**C**) Hepatic superoxide dismutase (SOD) activity (**D**) Hepatic glutathione peroxidase (GSH-Px) activity. Data are expressed as mean ± SEM, *n* = 6 mice per group. ## *p* < 0.01 vs. the control group. * *p* < 0.05 and ** *p* < 0.01 vs. the APAP group.

In order to study the protection mechanisms of ZJF on the hepatotoxicity induced by APAP in vivo, malondialdehyde (MDA), glutathione (GSH), superoxide dismutase (SOD) and glutathione peroxidase (GSH-Px) were used as indexes to evaluate the level of oxidative stress in liver. As a product of lipid peroxidation, MDA is frequently detected to evaluate the degree of oxidative injury. As shown in Figure 4A, there was a distinct increase of MDA content in the liver of mice exposed to APAP (*p* < 0.01), while pretreatment with ZJF (200 and 400 mg/kg) significantly reduced the MDA levels compared with the APAP treated group (*p* < 0.01). GSH and GSH-Px (Figure 4B,D) were depleted after the APAP administration when compared to the control (*p* < 0.01). However, GSH and GSH-Px were reversed with pretreatment of ZJF in a dose-dependent manner. The down-regulated activities of SOD after APAP poisoning were significantly elevated by ZJF at a dose of 200 and 400 mg/kg (Figure 4C). All the results above suggest that ZJF had a marked antioxidant activity and could suppress APAP-induced oxidative liver injury.

#### 2.3.3. Measurement of Nitric Oxide, Tumor Necrosis Factor-α, Interleukin-6 and Interleukin-1β Levels in Serum

**Figure 5:**
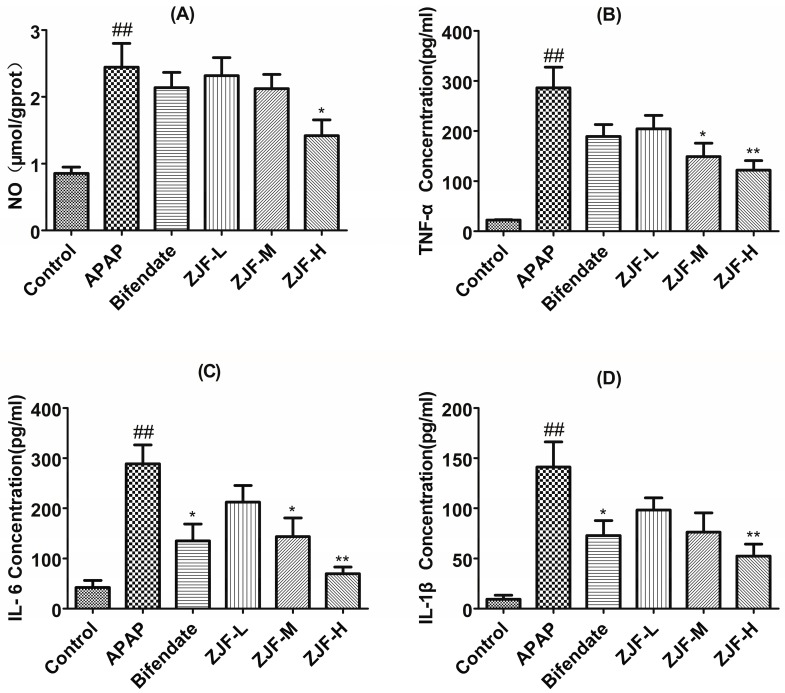
Preventive effects of different doses of ZJF on Nitric oxide (NO), tumor necrosis factor-α (TNF-α), interleukin-6 (IL-6), and interleukin-1β (IL-1β) levels in APAP-induced hepatotoxicity mice. (**A**) level of NO; (**B**) level of TNF-α; (**C**) level of IL-6; (**D**) level of IL-1β. Data are expressed as mean ± SEM, *n* = 6 mice per group. ## *p* < 0.01 vs. the control group. * *p* < 0.05 and ** *p* < 0.01 vs. the APAP group.

Nitric oxide (NO) is a highly reactive oxidant produced in the liver in response to inflammatory stimuli and plays a crucial role in hepatic injury [37]. Tumor necrosis factor-α (TNF-α), Interleukin-6 (IL-6), and Interleukin-1β (IL-1β) are the essential proinflammatory cytokines involved in the progression of APAP-induced liver injury [1,38]. As shown in Figure 5, APAP tremendously induced the elevation of NO production. Nevertheless, the NO level of the treatment group with a high dose of ZJF (400 mg/kg) was significantly lower than the APAP group (*p* < 0.05). TNF-α, IL-6 and IL-1β levels in the APAP group were markedly enhanced compared to the control group (*p* < 0.01), ZJF suppressed the elevation of these cytokines induced by the acute toxic dose of APAP in a dose-dependent manner. TNF-α and IL-6 levels were significantly different between ZJF (200 and 400 mg/kg) and the APAP group (*p* < 0.05 or *p* < 0.01).

#### 2.3.4. Effects of ZJF on the Protein Expression of NF-κB p65, Nrf2 and NQO1

**Figure 6:**
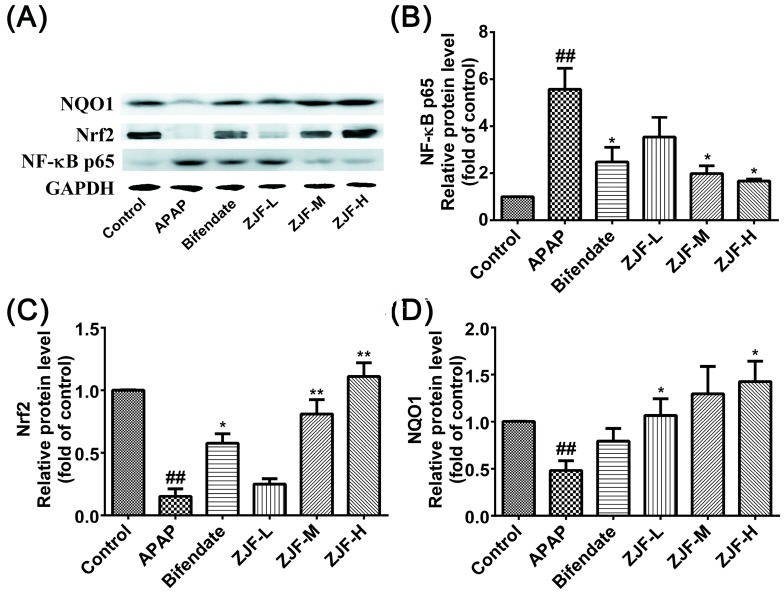
The effect of ZJF pretreatment on the nuclear factor kappa B p65 (NF-κB p65), nuclear factor erythroid 2-related factor 2 (Nrf-2), and NAD(P)H: quinone oxidoreductase 1 (NQO1 ) protein expression in response to the APAP-induced hepatotoxicity. (**A**) Western blotting analyses of NF-κB p65, Nrf-2, and NQO1 proteins; quantitative densitometric analyses of (**B**) NF-κB p65, (**C**) Nrf-2, and (**D**) NQO1 proteins normalized against glyceraldehyde-3-phosphate dehydrogenase (GAPDH). Each value represents the mean ± SEM of three independent experiments. ## *p*< 0.01 vs. the control group. * *p* < 0.05 and ** *p* < 0.01 vs. the APAP treated group.

Nrf2 plays an important role in cellular redox balance and serves as a major regulator of cellular defense associated pathways [39]. NF-κB P65 is associated with inflammatory responses in many diseases. Thus, to examine whether ZJF exerts its protective effect via NF-κB and Nrf2 pathways, we investigated NF-κB p65, Nrf2, and NAD(P)H: quinone oxidoreductase 1 NQO1 protein expression by Western blotting analysis. NF-κB p65 was up-regulated in response to the APAP-induced liver injury (Figure 6A,B), whereas this inflammatory mediator expression was alleviated by ZJF pretreatment in a dose-dependent manner. As shown in Figure 6C,D, APAP exposure caused a significant decrease in Nrf2 and NQO1 levels compared to the control group (*p* < 0.01). In contrast, ZJF up-regulated the expression of those proteins in a dose-dependent manner. The medium- and high-dose ZJF pretreatments significantly attenuated the decrease of Nrf2 expression (*p* < 0.01). The level of NQO1 protein showed more significant differences when pretreated with ZJF at low and high doses, compared to the APAP group (*p* < 0.05). Furthermore, at the same dose (200 mg/kg), ZJF affected the expression of Nrf2 and NQO1 protein more effectively than bifendate.

#### 2.3.5. Histopathological Evaluation

**Figure 7:**
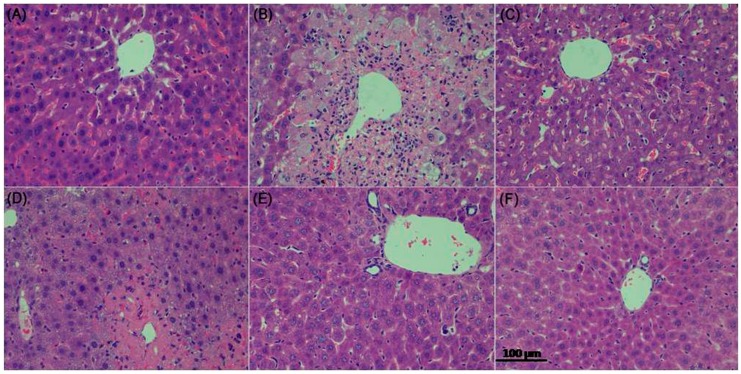
Histological analysis of the liver tissue of mice (HE staining 200×). (**A**) Control group; (**B**) APAP group; (**C**) Bifendate group; (**D**) ZJF-L group; (**E**) ZJF-M group and (**F**) ZJF-H group.

Hematoxylin and eosin stained sections are shown in Figure 7. Histology of liver tissues from the control mice showed a normal liver lobular architecture with cords of hepatocytes radiating from the central vein. Each cell exhibited a centrally located nucleus, but some binucleated cells were also seen (Figure 7A). In contrast, the APAP-intoxicated treatment exhibited severe histopathological changes such as swollen centrilobular hepatocytes with highly vacuolated cytoplasm, large areas of extensive cell necrosis with loss of hepatocyte architecture, and massive mononuclear cell infiltration in the portal area (Figure 7B). The histological examination of tissue sections from the mice pretreated with ZJF or bifendate showed the improvement of liver morphology. A low dose of ZJF slightly diminished the injured area, necrotic cells, and inflammatory infiltration compared to the control group (Figure 7D). Moreover, liver sections of the mice treated with medium- and high-dose ZJF exhibited more normal cellular architecture, disappearance of inflammatory infiltration, and patches of necrotic hepatocytes (Figure 7E,F).

#### 2.3.6. Immunohistochemistry Analysis

**Figure 8:**
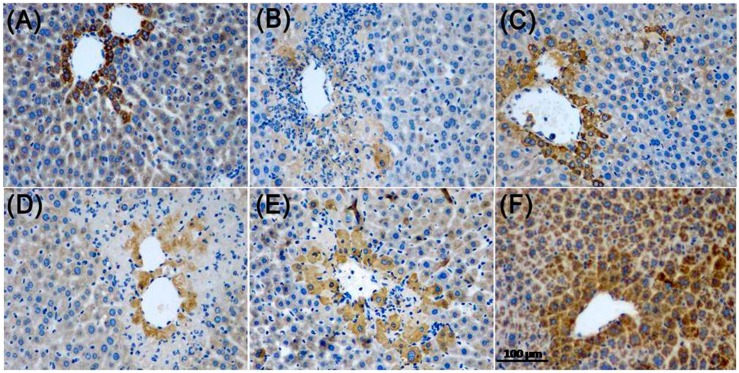
Effect of ZJF on Nrf2 expressions in the APAP-induced liver injury in mice. Photomicrographs were taken at 200×. (**A**) Control group; (**B**) APAP group; (**C**) Bifendate group; (**D**) ZJF-L group; (**E**) ZJF-M group and (**F**) ZJF-H group.

**Figure 9:**
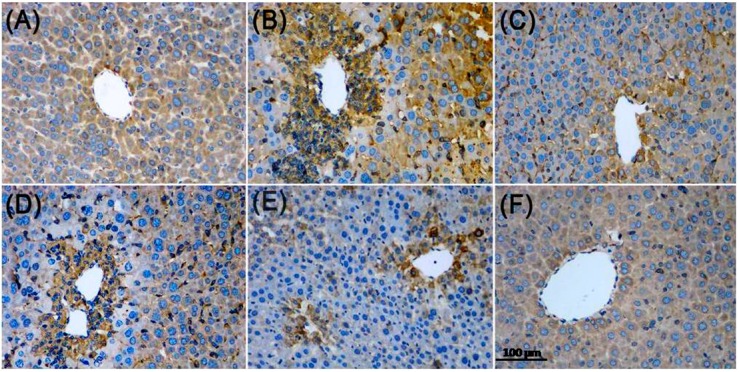
Effect of ZJF on nuclear factor NF-κB p65 expressions in the APAP-induced liver injury in mice. Photomicrographs were taken at 200×. (**A**) Control group; (**B**) APAP group; (**C**) Bifendate group; (**D**) ZJF-L group; (**E**) ZJF-M group and (**F**) ZJF-H group.

To further investigate the potential role of Nrf2 and NF-κB p65 in APAP-induced liver injury, we analyzed the protein expression of Nrf2 and NF-κB p65 in liver tissue by immunohistochemical staining. As shown in Figure 8, the accumulation of Nrf2 nuclear translocation was significantly lower in the control group, while ZJF reversed the suppression of Nrf2 nuclear translocation in the liver damage mice. The results in Figure 9 show that NF-κB activity was markedly induced in the hepatic cells of the APAP-treated mice, and the increased activity of NF-κB was inhibited by pretreatment with ZJF. These results imply that the protective effect of ZJF might be due to its antioxidant and anti-inflammatory abilities via the NF-κB and Nrf2 pathways.

## 3. Discussion

The jujube (*Ziziphus jujuba* Mill.), a hardy rhamnaceous plant, is widely used as a herb in traditional Chinese medicine [12]. It has gained a great deal of attention for its high nutritional value and multiple pharmacological effects [40]. Although there have been many pharmacological studies on jujube, the objective of the present study was to investigate ZJF’s main active ingredients and mechanisms associated with its hepatoprotective activity.

In this study, we isolated ZJF from *Ziziphus jujuba cv. Jinsixiaozao* by the macroporous resin adsorption method. Notably, total flavonoid content in ZJF was determined to be 40.85%. Furthermore, the chemical profile of ZJF was determined by LC-MS/MS analysis. Two kaempferol derivatives and seven quercetin derivatives were identified in ZJF. Particularly, quercetin derivatives are the main flavonoids found in ZJF. Numerous studies have shown that kaempferol and quercetin derivatives have a wide range of pharmacological activities [41,42]. The identification of the chemical composition in ZJF provided a basis for the further research of the pharmacological activity of ZJF.

In the assessment for antioxidant activity of ZJF, it showed a dose-dependent scavenging capacity against DPPH and ABTS radicals. Besides, ZJF exhibited a strong redox activity according to the ferric reducing antioxidant power assay. As a result, the ZJF was demonstrated to have a notable antioxidant activity in vitro and to have potential for antioxidant supplementation to protect humans against oxidative stress.

Due to its clinical relevance and experimental convenience, the APAP-induced liver injury model is widely used for evaluating the therapeutic potential of compounds purported to be hepatoprotective [43]. The APAP-induced hepatotoxicity is characterized as increased oxidative stress, mitochondria, and cell death [6]. Evidence indicates that the reactive metabolite of APAP initiates hepatocyte damage and inflammatory innate immune responses, which lead to the exacerbation and progression of liver injury [44]. Compared to the few studies reported on the hepatoprotective activity of ZJF, our study is the first to show that ZJF has a preventative effect on APAP-induced liver injury.

Hepatic damage can be evaluated by monitoring sensitive serum biochemical markers such as AST, ALT, TB, and ALP, which exude from the hepatocytes into the circulatory system because of altered membrane permeability [45]. Based on the test results, when mice were subjected to the APAP intoxication, there were a remarkable increase in AST, ALT, TB, and ALP levels compared with the control group. However, these reduced serum biochemical markers were significantly reduced by pretreatment with ZJF and bifendate, indicating that ZJF could preserve the structural integrity of hepatocytes and alleviate APAP-induced liver damage. These findings were also confirmed by histological observation. The APAP-challenged mice showed hepatocellular necrosis or apoptosis, inflammatory cell infiltration, cords of degenerated hepatocytes around the central vein, and other histological manifestations, while pretreatment of ZJF mitigated these changes.

APAP overdose induced the oxidative stress response in the liver, as shown in this study. In order to understand the antioxidant activities of ZJF in vivo, we determined the activities of antioxidant enzymes (SOD and GSH-Px) as well as the levels of GSH and MDA in the liver. SOD catalyzes the conversion of the superoxide radical into hydrogen peroxide, and then hydrogen peroxide is converted into oxygen and water by GSH-Px, generating reduced GSH as its substrate [46]. As a non-enzymatic antioxidant, GSH can remove superoxide ions and free radicals from the body to protect the integrity of cell membranes and maintain metabolism by anti-lipid oxidation [47]. MDA is generally considered to be an important marker of lipid peroxidation. In line with these concepts, we found that the activities of the major scavenger enzymes (SOD and GSH-Px) and the content of GSH were significantly decreased in the livers of the APAP-treated mice, while the level of MDA was elevated compared with the control group. As expected, the administration of ZJF markedly enhanced the levels of hepatic SOD, GSH-Px, GSH and inhibited the increase of MDA induced by APAP. Therefore, this study suggested that ZJF effectively protected the liver by improving enzymatic and non-enzymatic antioxidant defense systems against APAP-induced injury.

Moreover, Nrf2 is a major regulator of intracellular antioxidants. Under physiological conditions, Nrf2 binds to kelch-like ECH-associated protein-1 (Keap1) in the cytoplasm. When the body is subjected to oxidative stress, Nrf2 dissociates form Keap1 and translocates into the nucleus from cytosol and interacts with antioxidant response element (ARE), resulting in upregulating the expression of cytoprotective target genes including antioxidant enzymes and phase II detoxifying enzymes [48]. With ZJF intervention, our Western blotting and immunohistochemical analyses showed that Nrf2 protein expression and nuclear translocation in hepatic tissue were effectually increased compared with the APAP group. In addition, as one of phase II detoxifying enzymes, the NQO1 protein expression was also up-regulated compared with the control group. These results imply that ZJF treatment provided a hepatoprotective effect by activating the Nrf2 pathway.

Inflammation is closely interrelated with oxidation in the biological systems, and also plays a vital role in APAP-induced acute liver injury [1]. Inflammation occurs with the release of pro-inflammatory mediators, such as NO, TNF-α, IL-6 and IL-1β. Our study indicated that the levels of NO, TNF-α, IL-6, and IL-1β were elevated in the APAP-induced mice. ZJF administration dramatically decreased the expression of these pro-inflammatory mediators. NF-κB p65 is a key transcription factor in up-regulating inflammatory cytokines. In our study, ZJF prevented APAP-induced liver injury by reducing the protein expression of NF-κB p65, as evidenced by Western blotting and immunohistochemical staining. These results indicate that ZJF inhibited inflammatory responses initiated by APAP.

In summary, our present study demonstrated that ZJF possessed a protective action against APAP-induced liver injury. The underlying mechanisms of its hepatoprotective activity might involve the antioxidant defense through the Nrf2 pathway and anti-inflammatory activity via inhibiting NF-κB. These encouraging findings would be helpful for developing a potential hepatoprotective agent for remedying APAP-induced liver injury.

## 4. Materials and Methods

### 4.1. Materials and Chemicals

*Ziziphus jujuba cv. Jinsixiaozao* was obtained from Laoling, Shandong, China. Quercetin-3-*O*-galactoside, quercetin-3-*O*-rutinoside, and quercetin-3-*O*-glucoside were purchased from the National Institutes for Food and Drug Control (Beijing, China). Kaempferol 3-*O*-rutinoside and quercetin-3-*O*-rhamnoside were purchased from Chengdu Preferred Biotechnology Co. Ltd. (Chengdu, China). HPLC grade methanol and acetonitrile were purchased from Tedia Co. Inc. (Fairfield, CT, USA). Ultrapure water was prepared using a Milli-Q water purification system (Millipore, Bedford, MA, USA). APAP was produced by Aladdin chemistry Co. Ltd. (Shanghai, China) Anti-NF-κB p65 and Anti-NQO1 antibodies were purchased from Cell Signaling Technology (Danvers, MA, USA). Anti-Nrf2 antibodies were provided by Santa Cruz Biotechnology (Santa Cruz, CA, USA). Anti-GAPDH antibodies were provided from Proteintech Biotechnology (Rocky Hill, CT, USA). Other reagents were of analytical grade (Sinopharm Chemical Reagent Co. Ltd., Shanghai, China).

### 4.2. Animals

Male BALB/c mice (20–22 g) were purchased from Center for New Drug Evaluation of Shandong University, Jinan, China. Mice were housed in groups in ventilated cages, at a controlled temperature and humidity with a light/dark cycle of 12 hr. They were maintained with laboratory chow and water ad libitum. Mice in this experiment were acclimatized under laboratory condition for at least 1 week prior to the experiments. All procedures and care were in compliance with “Guide to the Care and Use of Laboratory Animals” (National Research Council and Shandong University) and approved by the Ethical Committee of Experimental Animal Center of Shandong University (No. 2016020, Shandong University, Jinan, China).

### 4.3. Preparation of the Flavonoids from Ziziphus jujuba cv. Jinsixiaozao

After removing the kernel, the jujube fruits were pulverized to homogeneous powder. A sample of 500 g powder was macerated with 70% aqueous ethanol for 3 h, then placed in ultrasonic cleaning bath and sonicated for 40 min. The extract was filtered and the filter cake was re-extracted by steps under the same conditions three times. The combined extracts were concentrated under reduced pressure in a rotary evaporator at 40 °C and a viscous mass was obtained after freeze drying. This mass was suspended in distilled water and chromatographed on an AB-8 macroporous absorption resin column (80 cm × 4.0 cm id.). The column was firstly eluted with distilled water. Subsequently, the highly polar compounds were removed with 3 BV of 10% aqueous ethanol. Finally, 70% aqueous ethanol was employed to rinse the column and the elution of 70% aqueous ethanol was collected, concentrated and then freeze-dried (3.22 g) for the following experiments.

### 4.4. Determination of Total Flavonoids Content

The total flavonoids content was determined using a colorimetric method [49]. Briefly, appropriately diluted sample (0.5 mL) was mixed with 5% (*w*/*v*) sodium nitrite (0.5 mL). Six minutes later, 10% (*w*/*v*) aluminum nitrate (0.5 mL) was added and incubated for 6 min. 4% (*w*/*v*) sodium hydroxide (4 mL) was added in the order stated. The final volume was adjusted to 10 mL with ethanol. The mixture was thoroughly mixed and allowed to stand for 15 min. Absorbance was measured at 510 nm against a blank solution using a UV spectrophotometer. The amount of the total flavonoids was expressed as rutin equivalents (mg rutin/g sample) through the calibration curve of rutin.

### 4.5. Separation and Identification of Flavonoids in ZJF

Analysis of the chemical composition of ZJF was performed on an Agilent 1260 HPLC system equipped with a diode array (Agilent Technologies, Palo Alto, CA, USA). Separation was carried out using a Gemini-NX C18 (4.6 mm × 250 mm, 5 µm, Phenomenex, Torrance, CA, USA) column at 30 °C, and the injection volume was 10 μL. The mobile phase consisted of solvent A (0.1% formic acid in water) and B (0.1% formic acid in acetonitrile) with gradient elution as follows: 0–15 min (15% B); 15–20 min (15–18% B); 20–30 min (18–20% B); 30–35 min (20–50% B); 35–40 min (50–15% B). The flow rate was set at 1.0 mL·min^−1^. Peaks of flavonoids were monitored at 350 nm, and UV/vis spectra were measured over a wavelength range of 200–400 nm in steps of 2 nm.

LC-MS/MS analysis was conducted on a Shimadzu LC-20AD XR series HPLC system (Shimadzu, Kyoto, Japan) coupled to an API 5000 electrospray triple-quadrupole tandem mass spectrometer (ESI-MS/MS) (Applied Biosystems/MDS SCIEX, Foster City, CA, USA). Compounds were applied on a Gemini-NX C18 (4.6 mm × 250 mm, 5 µm) column to separate flavonoids using the applied chromatographic conditions as same as those in the HPLC analysis. The LC eluate was introduced into the mass spectrometer from 5 to 40 min. Q1 MS (Q1) and Product Ion (MS2) analyses were operated in the negative-ion mode. Full scan data were acquired by scanning from the mass range of *m*/*z* 200 to 1000. The operational parameters of the mass spectrometer are summarized as follows: Nitrogen was used as the ion source gas, curtain gas and collision gas for API5000. Curtain gas (CUR), 15 psig; Ion source gas1 (GS1), 50 psig; Ion source gas2 (GS2), 50 psig; Ionspray voltage, −4500; Desolvation temperature, 450 °C; Declustering potential (DP) and entrance potential (EP) were set at −150 V and −10 V, respectively. For Product Ion (MS2) spectrometric procedures, collision energies (CE) were optimized to detect the fragments of these compounds.

Structural identification was performed by comparing retention time, UV absorption spectra, and mass spectral analysis with either the known authentic commercial standards available in our laboratory, or data reported in the peer-reviewed literature.

### 4.6. Antioxidant Activity In Vitro

#### 4.6.1. DPPH Radical Scavenging Assay

DPPH radical scavenging activity was evaluated according to a previously reported protocol [24], with slight modification. Briefly, samples (1.0 mL) at different concentrations were mixed with 0.15 mmol/L DPPH (3.0 mL), and then shaken vigorously. The absorbance value was read against a blank at 519 nm using a spectrophotometer after standing for 1 h at room temperature. All samples were carried out in triplicate with BHT and VC as references.

#### 4.6.2. ABTS Radical Scavenging Assay

ABTS scavenging ability was assessed according to a previously described method [50], with some modifications. In specific, ABTS+ was prepared by mixing ABTS stock solution (5 mL, 7 mM in pH 7.4 phosphate buffer solution) with 2.45 mM potassium persulfate (5 mL). The reaction mixture was kept in the dark at room temperature for 16 h. The mixture was then diluted with phosphate buffer solution (pH 7.4) to obtain an absorbance value of 0.700 ± 0.02 at 734 nm. Samples (20 μL) at various concentrations and ABTS+ solution (180 μL) were added to 96-well plates and mixed. The absorbance was recorded at 734 nm after a 6 min interval with a blank solution as reference.

#### 4.6.3. Reducing Power Assay (FRAP)

The reducing power assay (FRAP), was performed according to the method described by Huang et al. [51]. In brief, sample (1 mL) was mixed with 0.2 mM sodium phosphate buffer (2 mL, pH 6.6) and 1% potassium ferricyanide (2 mL). After incubating at 50 °C for 20 min, 10% (*w*/*v*) trichloroacetic acid (2 mL) were added. Subsequently, the reaction solution (2 mL) was combined with deionised water (2 mL) and 0.1% ferric chloride (0.4 mL). The mixture was allowed to stand for 30 min in the dark. Thereafter, the absorbance was measured at 700 nm. The increased absorbance of the reaction mixture indicated an increase in reducing power. Samples of BHT and VC at the same concentrations were used as positive control.

### 4.7. In Vivo Hepatoprotective Activity

#### 4.7.1. Drug Administration

In the experiment, acute liver injury was induced by giving an intraperitoneal injection of a sublethal dose of APAP (350 mg/kg). Based on the preliminary study, the ZJF concentration range of 100–400 mg/kg was selected for the study in vivo. Mice were fasted overnight for approximately 12 h before administration of APAP. Animals were divided into six groups as follows: (1) The normal control (Control); (2) APAP treated group (350 mg/kg, single dose) (APAP); (3) Bifendate (200 mg/kg) + APAP (350 mg/kg) (Bifendate); (4) ZJF (100 mg/kg) + APAP (350 mg/kg) (ZJF-L); (5) ZJF (200 mg/kg) + APAP (350 mg/kg) (ZJF-M); (6) ZJF (400 mg/kg) + APAP (350 mg/kg) (ZJF-H). ZJF and bifendate were suspended in 0.3% (*w*/*v*) sodium carboxy methyl cellulose (CMC-Na) for intragastric gavage. Mice were pre-treated with ZJF (100, 200, 400 mg/kg per day) for 10 consecutive days. On the last day, mice were given a single dose of APAP (350 mg/kg) by intraperitoneal injection after administration of ZJF for 1 h. The bifendate group was administrated in the same manner. Mice in control group and APAP-treated group were administered with 0.3% CMC-Na solution and injected intraperitoneally with normal saline and a single dose of APAP (350 mg/kg) after 1 h from the last administration, respectively. Mice were sacrificed at 16 h after APAP administration. Blood samples were collected from the eyeballs and liver tissues were removed immediately. Serum was separated by centrifugation at 4000 r/min for 15 min. A small portion of the liver was immediately fixed in 4% buffered paraformaldehyde and the remaining tissues were flash frozen in liquid nitrogen and stored at −80 °C for further use.

#### 4.7.2. Measurement of Serum Biochemical Parameters Related to Hepatic Dysfunction

Serum ALT, AST, ALP and TB were determined using commercial assay kits (Nanjing Jiancheng Bioengineering Institute, Nanjing, China) according to the manufacturer’s protocols.

#### 4.7.3. Measurement of GSH, MDA, SOD and GSH-Px in Liver Homogenate

Liver homogenates (10%, *w*/*v*) were prepared for evaluating the levels of GSH, MDA, SOD, and GSH-Px. All procedures were carried out according to the guidelines of the commercial assay kits (Nanjing Jiancheng Bioengineering Institute, Nanjing, China).

#### 4.7.4. Test for NO, TNF-α, IL-6 and IL-1β Levels in the Serum

Levels of serum TNF-α, IL-6, IL-1β were measured with mouse cytokines enzyme linked immunosorbent assay (ELISA) kit (MultiSciences Biotech Co., Ltd., Hangzhou, China) following the instructions of the manufacturer. Nitric oxide formation was measured by the commercial assay kits (Nanjing Jiancheng Bioengineering Institute, Nanjing, China) according to the manufacturer’s protocol.

#### 4.7.5. Western Blot Analysis for NF-κB p65, Nrf2, and NQO1

The mice liver tissues were homogenized in ice-cold radio immunoprecipitation assay (RIPA) lysis buffer (SolarbioLife Sciences) containing 1 mmol/L phenylmethylsulfonyl fluoride (PMSF, Sigma Aldrich, St. Louis, MO, USA) and 1% cocktail protease inhibitors (Sigma). Proteins were extracted by centrifugation and determined by a BCA kit (Beyotime Institute of Biotechnology, Beijing, China). Equal quantities of proteins were separated on 8–12% sodium dodecyl sulfate-polyacrylamide gel electrophoresis (SDS-PAGE) and then transferred onto a polyvinylidene difluoride (PVDF) membrane (Millipore Corp., Bedford, MA, USA) by electroblotting. The membranes were blocked in 5% skim milk for 2 h and subsequently probed overnight with the primary antibodies (1:1000) at 4 °C. After washing in TBST buffer, the membranes were exposed to HRP-conjugated secondary anti-rabbit or anti-mouse antibodies (1:5000) for 1 h at room temperature. Final detection was performed by enhanced chemiluminescence system (Merck Millipore, Darmstadt, Germany). The signal intensity of each band was quantified using the Alphalmager HP system (Cell Biosciences, Inc., Santa Clara, CA, USA). The relative optical densities of the bands were quantified using AlphaView SA software (Cell Biosciences, Inc., Santa Clara, CA, USA). All Western blot analyses were carried out at least three times.

#### 4.7.6. Histological Examination

Hematoxylin and eosin (H&E) staining was performed to evaluate cell necrosis. Liver tissue specimens were fixed in 4% paraformaldehyde solution for at least 24 h, processed routinely, embedded in paraffin and cut into 4 μm sections. Then these sections were deparaffinized and stained with hematoxylin and eosin (H&E) to evaluate morphology.

#### 4.7.7. Western Blot Analysis for NF-κB p65, Nrf2 and NQO1

Immunohistochemistry examination (IHC). Paraffin-embedded sections with a thickness of 4 μm were deparaffinized, rehydrated, and blocked in normal goat serum. The sections were incubated with NF-κB and Nrf2 antibodies overnight at 4 °C, followed by binding with a horseradish peroxidase-conjugated secondary antibody. Subsequently, immunoreaction was visualized by diaminobenzidine reaction and counterstained with hematoxylin.

### 4.8. Statistical Analysis

The experimental results were analyzed using the statistical software Prism version 6.0 (GraphPad Software, Inc., La Jolla, CA, USA). Data are presented as mean ± SD or mean ± SEM. The statistical significance of the difference between the control and treatment groups was assessed by either the Student *t*-test or ANOVA analysis for multiple comparisons.

## Figures and Tables

**Table 1:** Flavonoids identified by liquid chromatography–mass spectrometry/ mass spectrometry (LC-MS/MS) in flavonoids from ZJF.

Peak No.	Compounds	*t*_R_ (min)	λ_max_ (nm)	[M−H]^−^ (*m*/*z*)	Collision Energy (eV)	MS/MS (*m*/*z*)
1	Quercetin-3-*O*-robinobioside	17.63	252/353	608.9	−45	300.2
2	Quercetin-3-*O*-rutinoside	18.58	253/353	608.9	−45	300.0
3	Unidentified	19.35	247/355	671.5	−40	581.4/509.2
4	Quercetin-3-*O*-galactoside	20.25	253/353	463.3	−34	300.1
5	Quercetin 3-*O*-glucoside	21.27	253/353	463.3	−34	300.0
6	Kaempferol-3-*O*-robinobioside	22.41	260/350	593.4	−43	284.0
7	Kaempferol-3-*O*-rutinoside	25.50	258/350	593.2	−43	285.0
8	Unidentified	26.86	252/352	623.3	−46	315.1
9	Quercetin-3-*O*-arabino-rhamnoside	27.96	252/350	579.5	−41	300.0
10	Quercetin-3-*O*-rhamnoside	28.78	252/350	447.4	−32	300.1
11	Quercetin-3-*O*-xyloso-rhamnoside	29.67	255/350	579.5	−41	300.1
